# One-Step Assembly of a Porcine Epidemic Diarrhea Virus Infectious cDNA Clone by Homologous Recombination in Yeast: Rapid Manipulation of Viral Genome With CRISPR/Cas9 Gene-Editing Technology

**DOI:** 10.3389/fmicb.2022.787739

**Published:** 2022-02-10

**Authors:** Yanyang Zhou, Chenxi Li, Cicheng Ren, Jingbo Hu, Changxu Song, Xinjie Wang, Yanhua Li

**Affiliations:** ^1^College of Veterinary Medicine, Yangzhou University, Yangzhou, China; ^2^Comparative Medicine Research Institute, Yangzhou University, Yangzhou, China; ^3^Jiangsu Co-innovation Center for Prevention and Control of Important Animal Infectious Diseases and Zoonosis, Yangzhou, China; ^4^College of Animal Science & National Engineering Center for Swine Breeding Industry, South China Agriculture University, Guangzhou, China; ^5^Guangdong Key Laboratory of Mental Health and Cognitive Science, Center for Studies of Psychological Application, Institute for Brain Research and Rehabilitation, South China Normal University, Guangzhou, China

**Keywords:** porcine epidemic and diarrhea virus, transformation-associated recombination, reporter virus, infectious cDNA clone, yeast

## Abstract

Porcine epidemic diarrhea virus (PEDV), a swine enteric coronavirus causing acute diarrhea in piglets, is one of the major threatens to the pork industry globally. Reverse genetics is a valuable tool for the virological study and vaccine development for coronaviruses. Due to the large size and unstable problem in *Escherichia coli* of coronavirus genome, construction and manipulation of reverse genetics system for coronaviruses remain laborious and time-consuming. In this study, a reverse genetics system of the genotype II PEDV strain HM was generated using the transformation-associated recombination (TAR) technology in yeast within 1 week. The rescued virus (rPEDV) exhibited similar growth properties to the wild-type virus *in vitro*. With this PEDV infectious cDNA clone, CRISPR/Cas9 technology and homologous recombination were combined to generate a recombinant virus rPEDV-EGFP in which the ORF3 gene was swapped with an EGFP gene. The reporter virus displayed similar growth properties to the parental virus rPEDV and remained stable during serial passage *in vitro*. Of note, the strategies of construction and manipulation of PEDV infectious cDNA clone are extremely simple and efficient, which could be applied for other RNA viruses and DNA viruses.

## Introduction

Porcine epidemic diarrhea (PED) is a swine enteric disease caused by the porcine epidemic diarrhea virus (PEDV). It was first reported in the United Kingdom and Belgium in the 1970s (Wood, 1977) and then spread to European and Asian countries before 2010 (Song and Park, 2012). Since 2010, a highly virulent PEDV variant classified into genotype II (GII) emerged in China (Li et al., 2012; Sun et al., 2012) and the Chinese strain-like PEDV variants were also reported in the United States and other countries (Huang et al., 2013; Stevenson et al., 2013; Kochhar, 2014; Ojkic et al., 2015; Choudhury et al., 2016). The commercial vaccines developed with the classic PEDV strains are unable to provide full protection to those variants (Hou and Wang, 2019; Liu et al., 2020). PEDV variants have caused a significant economic loss and are still major threaten to the swine industry globally.

As a member of the genus *Alphacoronavirus* within the *Coronaviridae* family in the order *Nidovirales*, PEDV contains a single-stranded positive-sense RNA genome that is about 28 kb in length. The complete genome of PEDV encodes at least seven open reading frames (ORFs), including ORF1a, ORF1b, and ORF2–6. Two polyproteins encoded by ORF1a and ORF1b were further cleaved by viral proteases into 16 non-structural proteins (nsps), nsp1-nsp16. The ORF2–ORF6 encode four structural proteins, including spike (S) protein, envelop (E) protein, membrane (M) protein, and nucleocapsid (N) protein, and an accessory protein, ORF3 (Duarte et al., 1993). Of note, ORF3 was demonstrated to be non-essential for PEDV replication and can be replaced for the expression of reporter genes (Wang et al., 2012; Beall et al., 2016; Wongthida et al., 2017; Peng et al., 2020).

The reverse genetics system is one of the most critical tools in virological research, such as, study the functions of viral genes, viral replication and transcription mechanisms, virus–host interaction, and vaccine development through generation of recombinant viruses. To this end, four types of reverse genetics systems for PEDV have been reported, including a targeted RNA recombination method (Li et al., 2013), the infectious cDNA clones created by ligation of cDNA fragments into a bacterial artificial chromosome (BAC) one by one (Jengarn et al., 2015; Fan et al., 2017; Li et al., 2017; Peng et al., 2020), the infectious cDNA clones assembled by *in vitro* ligation of contiguous cDNA fragments (Beall et al., 2016), and the infectious cDNA clone based on the homologous recombination in yeast (Lu et al., 2020a). Those reverse genetics systems have been used to study the functions of several PEDV proteins and generate vaccine candidates *via* the modifications of viral proteins (Hou et al., 2017, 2019; Kaewborisuth et al., 2018; Kao et al., 2018; Deng et al., 2020; Lu et al., 2020a). To simplify the procedures to create PEDV mutants with defined genetic changes using infectious clones, the clustered regularly interspaced short palindromic repeats (CRISPR)/CRISPR-associated protein 9 (Cas9) technology was employed to cleave PEDV cDNA clone (Peng et al., 2020).

In this study, an infectious cDNA clone of GII PEDV strain HM was generated through one-step assembly in yeast through the TAR technology. With this infectious cDNA clone, a PEDV mutant expressing enhanced green fluorescence protein (EGFP) was created using CRISPR/Cas9 technology and *in vitro* homologous recombination. Here, we provided an efficient platform for the construction of PEDV infectious cDNA clone and manipulation of PEDV genome.

## Materials and Methods

### Cells, Viruses, and Antibodies

Vero CCL-81 cells (ATCC) were cultured in Dulbecco’s modified Eagle medium (DMEM) (HyClone) containing 10% fetal bovine serum (FBS) (Gemini Bio) and 1% penicillin–streptomycin (Gibco™). PEDV strain HM was isolated from the intestine sample of a neonatal piglet with acute diarrhea in China in 2016 using Vero CCL-81 cells supplemented with 10 μg/ml trypsin (Gibco™), and the passage 12 (P12) on Vero CCL-81 cells was used as seed stock in this study. A monoclonal antibody (mAb) against PEDV N protein was purchased from YouLong Biotech, and a mAb against β-tubulin was purchased from Bioworld.

### Determination of the Full-Length Genome of PEDV HM P12

The consensus sequence of the complete PEDV genomes was generated by multiple sequence alignment analysis using the CLC Genomics Workbench (QIAGEN) with all PEDV complete genomes in the GenBank sequence database. Seven pairs of primers targeting the highly conserved regions in the consensus sequence were designed to amplify the complete PEDV genome by reverse transcription PCR. The sequences of these primers are available upon request. Briefly, viral RNA of PEDV HM P12 was extracted with a viral DNA/RNA extraction kit (Vazyme Biotech) according to the manufacturer’s instructions. cDNA was generated with the Superscript™ IV first-strand synthesis system (Thermo Fisher Scientific) followed by viral RNA removal with RNase H (NEB). Seven overlapping fragments covering the PEDV HM P12 genome were PCR amplified using Q5 high-fidelity DNA polymerase (NEB). The PCR products were purified and sent for DNA sequencing by the GENEWIZ facility in Suzhou, China. Finally, the complete genome of PEDV HM P12 assembled with SnapGene was submitted to GenBank under accession No. MZ342899.

### In-yeast Assembly of a Full-Length cDNA Clone of PEDV HM P12

The pYES1L vector (Thermo Fisher Scientific) containing a yeast artificial chromosome (YAC) and a BAC was utilized to assemble an infectious cDNA clone of PEDV HM (P12) in yeast. With the YAC/BAC, this infectious cDNA clone will be able to replicate in both yeast and *E. coli*. We inserted the CMV early promoter, hepatitis delta virus (HDV) ribozyme sequence, and bovine growth hormone (BGH) termination signal into linearized pYES1L vector, generating the plasmid pYES1L-CMV-HDVrbz-bGH. The linearized pYES1L-CMV-HDVrbz-BGH vector was prepared by PCR amplification with Q5 high-fidelity DNA polymerase (NEB) using primers pYES1L-F and pYES1L-R. The cDNA of PEDV HM P12 was produced with the Superscript™ IV first-strand synthesis system (Thermo Fisher Scientific) followed by viral RNA removal with RNase H (NEB). With the viral cDNA, seven overlapping DNA fragments (F1: nt 1–4,020, primers YES1L-PEDV-F1/YES1L-PEDV-R1; F2: nt 3,986–8,107, primers YES1L-PEDV-F2/YES1L-PEDV-R2; F3: nt 8,062–12,071, primers YES1L-PEDV-F3/YES1L-PEDV-R3; F4: nt 12,034–16,836, primers YES1L-PEDV-F4/YES1L-PEDV-R4; F5: nt 16,807–20,223, primers YES1L-PEDV-F5/YES1L-PEDV-R5; F6: nt 20,191–24,495, primers YES1L-PEDV-F6/YES1L-PEDV-R6; F7: nt 24,455–28,064, primers YES1L-PEDV-F7/YES1L-PEDV-R7) covering the complete PEDV genome were amplified using Q5 high-fidelity DNA polymerase (NEB) according to the manufacturer’s instructions and then assemble with the linearized pYES1L-CMV-HDVrbz-BGH vector through transformation-associated recombination (TAR) in yeast. The overlapping regions (>30 nt) between neighboring DNA fragments enabled the homologous recombination in yeast. Briefly, the transformation of the MaV203 competent yeast cells (Thermo Fisher Scientific) with the mixture of 100 ng linearized vector and 200 ng of each F1–F7 DNA fragment was conducted with the PEG/LiAc solution according to the manufacturer’s instructions of the GeneArt® High-Order Genetic Assembly System (Thermo Fisher Scientific). Colony PCR was performed to screen yeast colonies containing the full-length cDNA clone pYES1L-PEDV using primer pairs, YES1L-PEDV-F1/PEDV-seq-R12 and PEDV-seq-F25/YES1L-PEDV-R7 (Table 1). The lysate of a positive yeast colony was further electroplated into DH10B competent *E. coli* cell to prepare pYES1L-PEDV plasmid using NucleoBond® Xtra Midi kit (MACHEREY-NAGEL). In addition, a plasmid pCAGGS-PEDV-N for ectopically expressing PEDV N was constructed by cloning N protein-coding sequence into pCAGGS vector using *SacI* and *XhoI* restriction enzyme sites.

**Table 1:** Oligonucleotides used in this study.

Name	Sequence (5'-3')
pYES1L-F	AAAAAAAAAAAAAAAAAAAAAAAAAAAAAAAAAGGCCG
pYES1L-R	CTATTAGAGCTCAAGCTCTGC
YES1L-PEDV-F1	TCTATATAAGCAGAGCTTGAGCTCTAATAGACTTAAAAGATTTTCTATCTACGG
YES1L-PEDV-R1	GTTGGTGACATAATGACCAC
YES1L-PEDV-F2	TTATAGGCAAGGATAGTGGTC
YES1L-PEDV-R2	CTATAGGACAACTAGGATTGTT
YES1L-PEDV-F3	GCCAAGTTTGGTTTCACC
YES1L-PEDV-R3	AACTTACGTGGTGGTTC
YES1L-PEDV-F4	GGTTGTAACACTATTGAGCTAGAACC
YES1L-PEDV-R4	TGCTTGAACCATTCTCCACCTGAACATTAC
YES1L-PEDV-F5	GTAATGTTCAGGTGGAGAATGGTTCAAGCA
YES1L-PEDV-R5	TACCACCAAGTGCCAAC
YES1L-PEDV-F6	GTGTCATCACTGAAAAGTTGG
YES1L-PEDV-R6	CGCTGCTCTAAATCTGC
YES1L-PEDV-F7	TATCTTAATCTCACTGGTGAAATTGC
YES1L-PEDV-R7	CCTTTTTTTTTTTTTTTTTTTTTTTTTTTTTTTTTGTGTATCCATATC
PEDV-N-F	TTCGAGCTCGCCACCATGGCTTCTGTCAGCTT
PEDV-N-R	TAGCTCGAGTTAATTTCCTGTATCGAAGATCTCG
PEDV-EGFP-F	AGTCGTCAAAGATGTCTCTAAGTCTATGGTGAGCAAGGGCG
PEDV-EGFP-R	GAATTGAGTCAAATGCAGCATTAGTAATGCCAACAATTTTACTTGTACAGCTCGTCCA
PEDV-seq-R12	ACTAAGCTTGGTAGTGC
PEDV-seq-F25	ACTGGTAATGCAAAACC
PEDV-sgRNA1	GACAGCGTCCGAAGACAAGTGTTTTAGAGCTAGAAATAGCAAGTTAAAATAAGGCTAGTCCGTTATCAACTTGAAAAAGTGGCACCGAGTCGGTGC
PEDV-sgRNA2	TTTTCGCAACATCAAATTGTGTTTTAGAGCTAGAAATAGCAAGTTAAAATAAGGCTAGTCCGTTATCAACTTGAAAAAGTGGCACCGAGTCGGTGC

### CRISPR/Cas9 Manipulation of pYES1L-PEDV and Swapping ORF3 With an EGFP Gene

A cDNA clone containing an EGFP gene was constructed by swapping a partial ORF3 coding sequence which is nt 24,856–25,395 of the PEDV HM genome. In brief, two guide RNAs, PEDV-sgRNA1 and PEDV-sgRNA2, synthesized by GenScript were used for CRISPR/Cas9 cleavage of the pYES1L-PEDV plasmid. The cleavage reaction containing 3 μg plasmid was conducted with *Streptococcus pyogenes* Cas9 nuclease (Vazyme Biotech) according to the manufacturer’s instructions. The linearized pYES1L-PEDV was verified by electrophoresis and purified with the Zymoclean Large Fragment DNA Recovery kit (ZYMO RESEARCH) per the manufacturer’s instructions. The EGFP gene amplified with primers EGFP-F and EGFP-R was inserted into the linearized pYES1L-PEDV through homologous recombination using HiFi DNA Assembly Master Mix (NEB) according to the manufacturer’s instructions. Two homologous arms between the EGFP gene and the linearized pYES1L-PEDV are around 20 bp in length. The recombinant plasmid was designated as pYES1L-PEDV-EGFP.

### Recovery of Recombinant Viruses

BHK-21 cells at ~80% confluence into a six-well culture plate were co-transfected with 0.5 μg pCAGGS-PEDV-N and 2 μg full-length cDNA clone, pYES1L-PEDV or pYES1L-PEDV-EGFP, using Lipofectamine® 3000 transfection reagent (Thermo Fisher Scientific) according to the manufacturer’s instructions. At 2 days post-transfection (dpt), culture supernatant was harvested to infect Vero CCL-81 cells in a six-well plate. After 2-h incubation, the cell monolayers were rinsed twice with 1xPBS and further cultured with 3 ml of DMEM supplemented with 10 μg/ml of trypsin (Thermo Fisher Scientific) in a 37°C, 5% CO_2_ incubator. Cells were observed daily for the appearance of cytopathic effect (CPE) or green fluorescence under the IX73 epifluorescence microscope (Olympus). Around 5 dpt, the culture supernatant was harvested as P0 virus and stored at −80°C and then further passaged on Vero CCL-81 cells for at least 10 times.

### Indirect Immunofluorescence Assay

Vero CCL-81 cells in 12-well culture plates were infected with PEDV HM, rPEDV, or rPEDV-EGFP for 24 h. The cell monolayers were fixed with 4% paraformaldehyde for 10 min and then permeabilized with 1xPBS containing 0.1% Triton X-100 for 10 min at room temperature. After 30 min blocking with 1xPBS containing 1% bovine serum albumin, the cell monolayers were washed thrice with 1xPBS and then incubated with mAb against PEDV N protein at 37°C for 1 h. After five washes, the cell monolayers were stained with TRITC-conjugated goat anti-mouse IgG(H + L) (Jackson ImmunoResearch Inc.). Cell nuclei were counterstained with 4′,6-diamidino-2-phenylindole (DAPI) solution (Solarbio life sciences) for 5 min at room temperature. After extensive washes with 1xPBS, fluorescent images were visualized with the IX73 epifluorescence microscope (Olympus).

### Western Blot Analysis

Vero CCL-81 cells seeded in 12-well plates were infected with PEDV HM P12 or rPEDV, and mock infection was used as control. At 24 h post-infection (hpi), cells were harvested with IP lysis buffer (Beyotime Biotechnology) supplemented with protease inhibitor cocktail (Roche). The cell lysates mixed with 5x loading buffer were boiled at 95°C for 5 min and separated by electrophoresis in 12% SDS-PAGE gel. The proteins in SDS-PAGE gel were transferred onto nitrocellulose membranes (Sangon Biotech). After 1 h blocking with 10% skimmed milk, the membranes were incubated with primary antibodies against PEDV N protein and β-tubulin, followed by HRP-conjugated secondary antibody, and visualized with ECL substrate (Vazyme Biotech) using the Tanon 5200 Multi imaging system.

### Identification of the Subgenomic mRNA Expressing EGFP by rPEDV-EGFP in Vero CCL-81 Cells

We confirmed that EGFP protein was expressed through an additional subgenomic RNA in Vero CCL-81 cells infected with the rPEDV-EGFP virus. Total cellular RNA was extracted from Vero CCL-81 cells infected with rPEDV-EGFP P5 virus using FastPure Cell/Tissue Total RNA Isolation Kit (Vazyme Biotech). The subgenomic RNA expressing EGFP was amplified with primers anchoring 5’UTR and EGFP using HiScript II One-Step RT-PCR Kit (Vazyme Biotech). The PCR product was purified and sent for DNA sequencing by GENEWIZ.

### Viral Growth Curve

The confluent Vero CCL-81 cells in 24-well culture plates were infected with PEDV HM, rPEDV, or rPEDV-EGFP at a multiplicity of infection (MOI) of 0.1. Cells were incubated with virus supernatant diluted with DMEM for 1 h, then washed twice with 1xPBS, and supplemented with 1 ml DMEM containing 10 μg/ml of trypsin (Thermo Fisher Scientific). Virus supernatants of two wells for each virus were harvested at 0, 24, 48, and 72 hpi) for virus titration by TCID_50_. The viral growth curves were created with GraphPad Prism 8.

### Plaque Assay

The confluent Vero CCL-81 cells in 12-well culture plates were infected with 10-fold diluted PEDV HM, rPEDV, or rPEDV-EGFP, respectively. Cells were incubated with virus supernatant diluted with DMEM for 2 h, then washed twice with 1xPBS, and then overlaid with 5 ml of DMEM containing 1% UltraPure™ Low Melting Point Agarose (Thermo Fisher Scientific) and 10 μg/ml trypsin (Thermo Fisher Scientific). At 3 days post-infection (dpi), the cells were fixed with 4% paraformaldehyde and stained with 0.1% crystal violet to visualize plaques.

## Results

### Full-Length Genome Sequence Analysis of PEDV HM Strain P12

**Figure 1:**
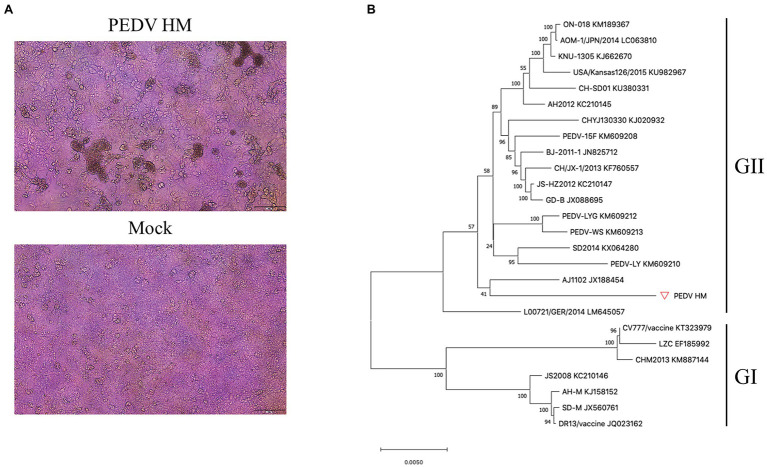
Isolation and phylogenetic analysis of the PEDV HM strain. **(A)** PEDV HM infection of Vero CCL-81 cells. Vero CCL-81 cells were inoculated with PEDV HM P12 at an MOI of 0.1 or mock-infected, and cell monolayers were monitored daily for CPE under a bright field microscope. **(B)** Evolutionary analysis of the full-length genomic sequences of PEDV HM strain and 25 representative PEDV strains. The phylogenetic tree was generated with the neighbor-joining method using MEGAX, and bootstrap values were set as 1,000 for each node. The evolutionary distances were computed using the Maximum Composite Likelihood method and are in the units of the number of base substitutions per site. The names of the strains, GenBank accession numbers, and genogroups are shown.

We isolated the PEDV HM strain through inoculation of Vero CCL-81 cells with an intestine sample from a piglet with acute diarrhea. This virus was further passaged 12 times on Vero CCL-81 cells. At 2 dpi, the typical CPE formation of PEDV infection was observed in Vero CCL-81 cells infected with the P12 virus (Figure 1A). To determine the complete genome of this virus, a panel of primers targeting the highly conserved regions of the PEDV genome was designed to amplify seven overlapping fragments covering the entire genome. These seven fragments were sequenced by the Sanger sequencing method, and the complete genome was assembled with SnapGene software and saved under the GenBank accession No. MZ342899. We further conducted a phylogenetic analysis with this genome and 25 representative PEDV genomes downloaded from the GenBank database using MEGAX (Kumar et al., 2018). Based on the phylogenetic tree generated with the neighbor-joining method, PEDV strain HM was clustered with GII PEDV strains (Figure 1B) which are new PEDV variants and shared 98.2% sequence identity with AJ1102 strain which was isolated from a neonatal piglet with acute diarrhea in China in 2011 (Bi et al., 2012).

### Construction of the Full-Length cDNA Clone of PEDV HM Strain P12

**Figure 2:**
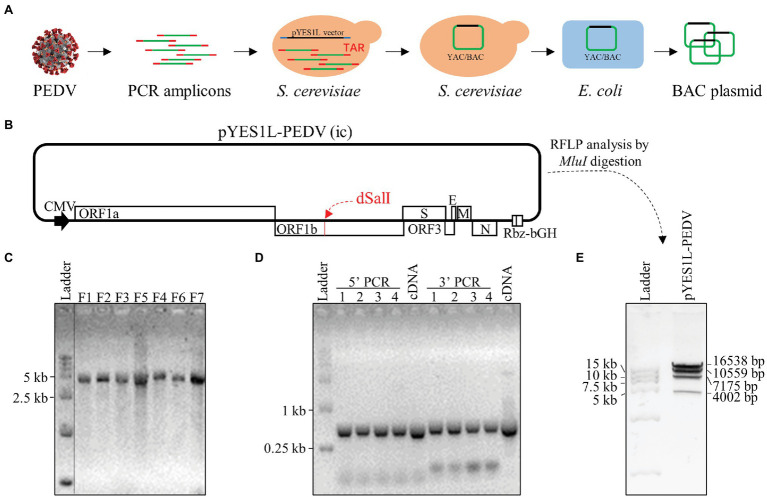
Construction of a PEDV infectious cDNA clone by homologous recombination in *yeast*. **(A)** The procedure of PEDV infectious cDNA clone assembly. Briefly, the complete PEDV genome was divided into seven fragments, and the neighboring fragments share at least 30 nucleotides overlapping regions. Together with a linearized vector (pYES1L) all seven cDNA fragments were transformed into *Yeast* MaV203 competent cells, and the full-length cDNA clone was assembled by transformation-associated recombination (TAR) in yeast. The yeast colonies were screened by colony PCR targeting the junction regions. The full-length cDNA clone was extracted from the positive colony and electroporated into DH10B *Escherichia coli*. Competent cells. Finally, the full-length PEDV cDNA clone in *E. coli*. Was purified using a Plasmid Midiprep Kit (MACHEREY-NAGEL). **(B)** A schematic diagram of PEDV infectious cDNA clone. In this YAC/BAC vector-based infectious cDNA clone, a CMV early promoter and a hepatitis D virus ribozyme (Rbz) followed by a BGH termination signal were added at 5' and 3' end of viral genomic cDNA, respectively. **(C)** Agarose gel electrophoresis of seven cDNA fragments covering the complete genome of PEDV HM strain. The complete genome of PEDV HM was amplified by RT-PCR with the Q5 high-fidelity DNA polymerase (NEB) using primers listed in Table 1. The gel-purified PCR products were separated in 1% agarose gel. **(D)** Colony PCR screening of positive clones. Five yeast colonies grew on CSM-Trp agar plate were picked for colony PCR using two pairs of primers (Table 1) targeting the junction regions of the 5' end and 3' end of the PEDV genome. **(E)** Restriction fragment length polymorphism analysis of the PEDV cDNA clone. The cDNA clone pYES1L-PEDV digested with *MluI* restriction enzyme was separated in 0.8% agarose gel. Four DNA fragments were observed at the predicted sizes of 16,538, 10,559, 7,175, and 4,002 bp.

To assemble the full-length cDNA clone of PEDV through the TAR technology (Figure 2A), we chose the pYES1L vector containing the YAC/BAC because of its replication ability in yeast and high stability in *E. coli*. To facilitate TAR in yeast, the pYES1L vector was initially modified to include the CMV early promoter, HDV ribozyme, and BGH termination signal, and designated as pYES1L-CMV-HDVrbz-BGH (Figure 2B). Through transformation with linearized pYES1L-CMV-HDVrbz-BGH into the MaV203 competent yeast cells, seven overlapping DNA fragments were amplified and assembled into pYES1L-PEDV (Figure 2C). In addition, to distinguish from wild-type virus, a genetic marker which is a *SalI* site knockout in ORF1b was created by incorporating two nucleotide substitutions in primers pYES1L-PEDV-F5 and pYES1L-PEDV-R4 (Figure 2B). The yeast colony containing pYES1L-PEDV was screened with colony PCR with primer pairs, YES1L-PEDV-F1/PEDV-seq-R12 and PEDV-seq-F25/YES1L-PEDV-R7 (Figure 2D). All five yeast colonies screened are positive, suggesting the superior efficiency of the TAR method in the assembly of a large viral genome. Then, one positive yeast colony was lysis to transform *E. coli*, and the infectious clone plasmid was prepared for verification and virus recovery. Restriction fragment length polymorphism by *MluI* digestion and DNA sequencing was conducted to verify the sequence of pYES1L-PEDV (Figure 2E). The results suggested that the full-length cDNA clone of PEDV HM strain P12 was successfully constructed, and no deletion or insertion compared with the wild-type sequence was identified.

### Recovery and *in vitro* Characterization of rPEDV

**Figure 3:**
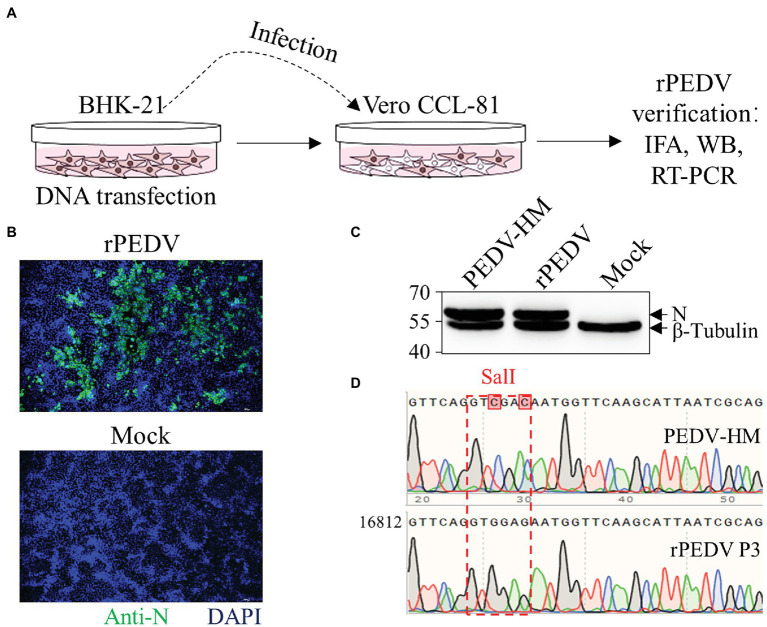
Recovery and verification of recombinant PEDV. **(A)** A schematic diagram of recombinant PEDV recovery and verification. In brief, BHK-21 cells at 70% confluence in a six-well plate were transfected with 2 μg PEDV infectious cDNA clone and 0.5 μg pCAGGS-PEDV-N using Lipofectamine 3000 transfection reagent according to the manufacturer’s instructions. At 48 h post-transfection (hpt), the culture supernatant harvested from BHK-21 cells was transferred to the confluent Vero CCL-81 cell monolayer and replenished with DMEM containing 10 μg/ml trypsin. The cell monolayer was checked daily for CPE, and the expression of N protein was evaluated by IFA and western blot analysis (WB). **(B)** N protein detection by IFA. At 4 days post-infection (dpi), Vero CCL-81 cells were fixed and stained with a mAb against N protein followed by incubation with the Alexa Fluor 488-conjugated goat anti-mouse IgG(H + L). The fluorescent pictures were taken under an IX73 epifluorescence microscope. **(C)** N protein detection by WB. Vero CCL-81 cells were infected with PEDV HM P12 or rPEDV at an MOI of 0.1, and mock-infected Vero CCL-81 cells were used as control. At 2 dpi, cell lysates harvested with IP lysis buffer were used for (WB) with a mAb against N protein, and β-tubulin was detected as a loading control. **(D)** Verification of recombinant PEDV by checking the genetic marker. The regions from nt 16,768 to 19,263 within PEDV HM P12 and rPEDV were amplified and verified by DNA sequencing. As expected, the *SalI* site in rPEDV was disrupted by two nucleotide substitutions at nt 16,820 and 16,823.

**Figure 4:**
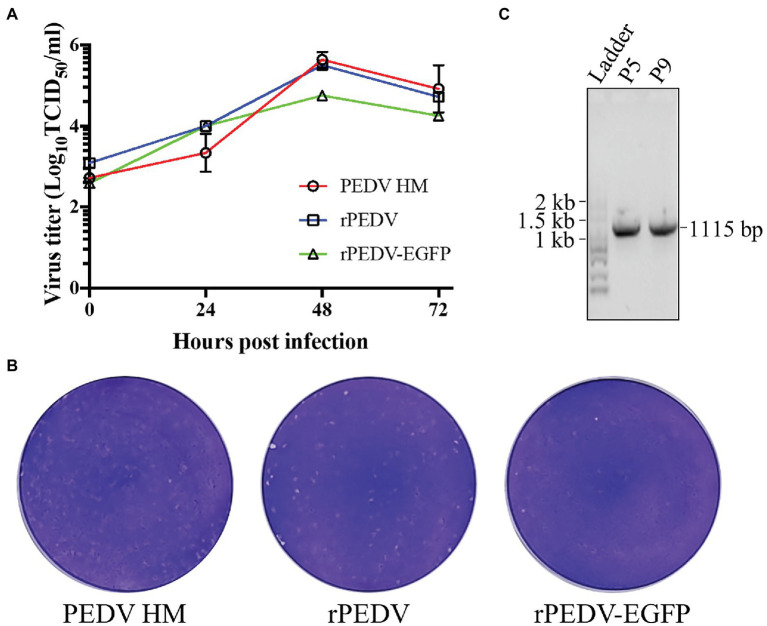
*In vitro* characterization of the recombinant PEDV. **(A)** The multiple-step growth curves. Vero CCL-81 cells were inoculated with PEDV HM P12, rPEDV P5, or rPEDV-EGFP P5 at an MOI of 0.1. Culture supernatants were harvested at 0, 24, 48, 72 hpi and titrated by TCID_50_. Each data point represents the mean value of two replicates with SD. **(B)** Plaque assay. The confluent Vero CCL-81 cells were infected with 10-fold diluted PEDV HM, rPEDV, or rPEDV-EGFP, respectively. Cells were overlaid with 5 ml of DMEM containing 1% UltraPure™ Low Melting Point Agarose (Thermo Fisher Scientific) and 10 μg/ml trypsin (Thermo Fisher Scientific). At 3 dpi, plaques were visualized by 0.1% crystal violet staining. **(C)** The stability of the EGFP gene in rPEDV-EGFP during *in vitro* passages. To this end, rPEDV-EGFP was serially passaged nine times in Vero CCL-81 cells. Viral RNAs of P5 and P9 were extracted and used for RT-PCR amplification of genomic regions containing the EGFP gene. The amplicons were separated in 1% agarose gel, and their expected size is 1,115 bp. DNA sequencing was conducted to confirm their sequences.

BHK-21 cells were co-transfected with pYES1L-PEDV and pCAGGS-PEDV-N to rescue the recombinant PEDV, and virus supernatant was further transferred to the confluent Vero CCL-81 cell monolayer (Figure 3A). At 3 dpi, the typical CPE characterized by cell fusion and syncytium formation was observed. The successful recovery of rPEDV was confirmed by indirect immunofluorescence assay (IFA) and western blot analysis (WB) with mouse mAb against PEDV N protein (Figures 3B,C). To rule to the possibility of contamination, the designed genetic marker which is the inactivation of the *SalI* site (nt 16,818–16,823) in ORF1b of rPEDV was verified by RT-PCR and DNA sequencing (Figure 3D). We further *in vitro* characterized the rescued rPEDV (P5) and the parental PEDV HM P12 by multiple-step growth curve and plaque assay using Vero CCL-81 cells. As shown in Figure 4A, rPEDV displayed similar growth behaviors to the parental virus, and both reached peak titers at 48 hpi. For the size and morphology, no significant difference was observed between the plaques formed in cells infected with the recombinant virus and the parental virus (Figure 4B).

### Construction of a PEDV cDNA Clone Expressing EGFP With ORF3 Swapped by CRISPR/Cas9 Technology and Homologous Recombination

**Figure 5:**
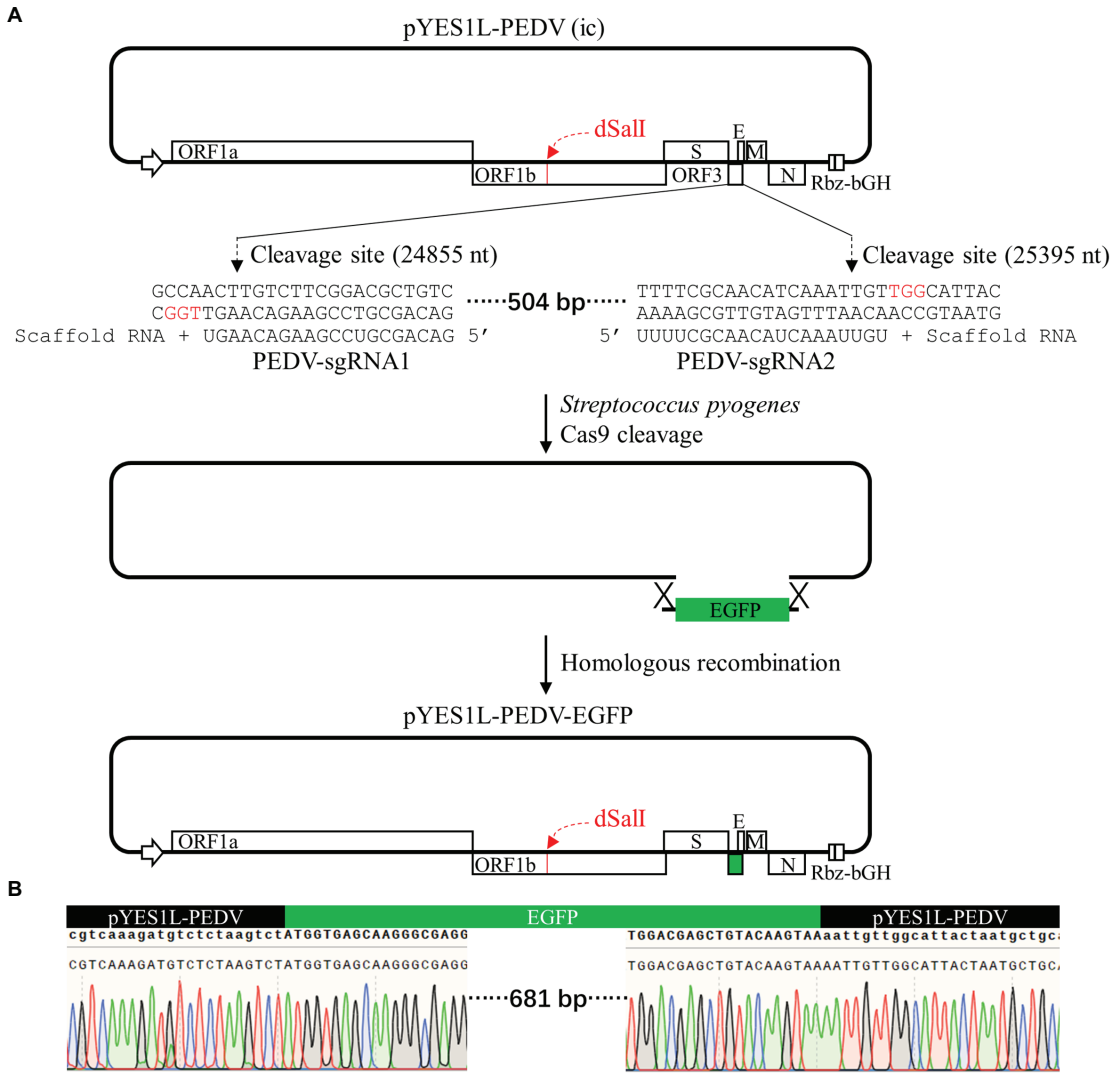
Construction of a porcine epidemic diarrhea virus (PEDV) cDNA clone expressing EGFP with ORF3 swapped. **(A)** The schematic diagram of CRISPR/Cas9 editing PEDV cDNA clone. pYES1L-PEDV was linearized using *Streptococcus pyogenes* Cas9 nuclease with two guide RNAs, PEDV-sgRNA1 and PEDV-sgRNA2. The homologous arms with this linearized cDNA clone backbone (>20 nt) at both terminals of the EGFP gene were introduced by primers EGFP-F and EGFP-R. This EGFP gene was assembled into a linearized cDNA clone backbone through homologous recombination using HiFi DNA assembly master mix (NEB), and the resulting plasmid was designated as pYES1L-PEDV-EGFP. **(B)** Verification of the EGFP insertion by DNA sequencing.

The ORF3 gene is non-essential for PEDV replication and can be replaced with reporter genes (Wang et al., 2012; Beall et al., 2016; Wongthida et al., 2017; Peng et al., 2020). To prove that our YAC/BAC-based PEDV infectious cDNA is convenient for genome manipulation, the CRISPR/Cas9 technology and homologous recombination technology were employed to construct the pYES1L-PEDV-EGFP variant clone in which the PEDV ORF3 gene was swapped with an EGFP gene. As shown in Figure 5A, two guide RNAs, PEDV-sgRNA1 and PEDV-sgRNA2, compatible with *S. pyogenes* Cas9 protein were used to cleave at the sites of 24,855–24,856 nt and 25,395–25,396 nt within the ORF3 gene, and the linearized pYES1L-PEDV was purified for homologous recombination *in vitro*. The EGFP gene containing at least 20 nt homologous arm at both terminals with linearized pYES1L-PEDV was amplified with primers, EGFP-F and EGFP-R (Table 1). The homologous recombination was conducted with the purified DNA fragments using HiFi DNA Assembly Master Mix (NEB; Figure 5A). The recombination reaction was electroporated into DH10B competent cells using Gene Pulser Xcell™ (Bio-Rad) according to the manufacturer’s instructions, and colony PCR amplifying the EGFP gene was performed to screen colonies containing pYES1L-PEDV-EGFP, and the positive clones were verified by DNA sequencing (Figure 5B). To our surprise, all five colonies tested by colony PCR are positive, which suggested the high efficiency of our strategy in the manipulation of the PEDV genome.

### Recovery and *in vitro* Characterization of rPEDV-EGFP

**Figure 6:**
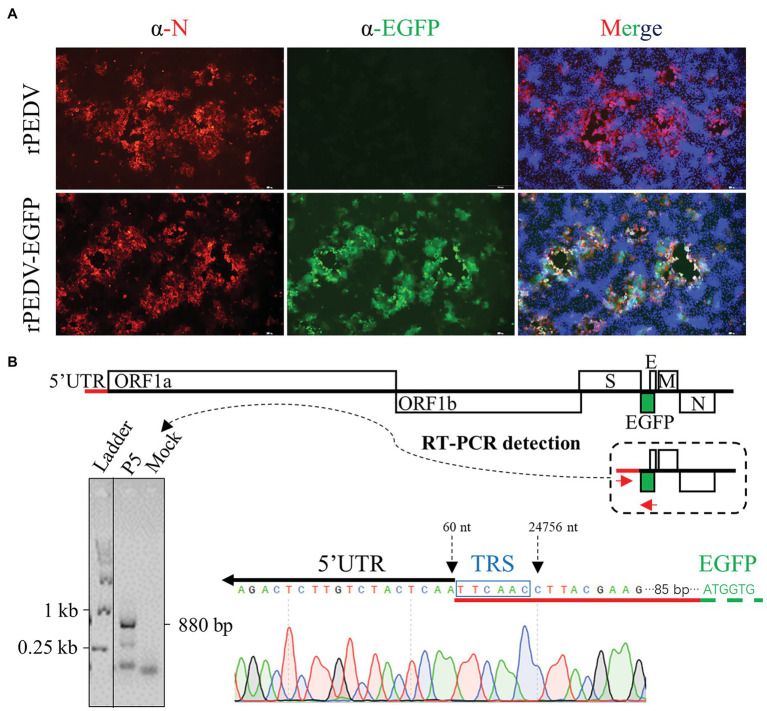
Recovery and verification of rPEDV-EGFP. **(A)** N protein expression detection by indirect immunofluorescence assay (IFA). Vero CCL-81 cells were infected rPEDV or rPEDV-EGFP at an MOI of 0.1. At 24 h post-infection (hpi), cells were fixed and stained with a mAb against PEDV N protein followed by incubation with TRITC-conjugated goat anti-mouse IgG(H + L). Cell nuclei were counterstained with DAPI. Pictures were taken under the IX73 epifluorescence microscope (Olympus). **(B)** Identification of the subgenomic RNA expressing EGFP in rPEDV-EGFP infected Vero CCL-81 cells. Vero CCL-81 cells infected with rPEDV-EGFP P5 at an MOI of 0.1 MOI or mock-infected were harvested with TRIzol reagent (Vazyme). Total cellular RNA was extracted and used for RT-PCR amplification of the subgenomic RNA expressing EGFP using primers YES1L-PEDV-F1 and EGFP-R. The expected size of this amplicon is around 880 bp in length. The body TRS-leader TRS junction was identified by DNA sequencing with the amplicon.

To rescue the recombinant virus, the culture supernatant of BHK-21 cells co-transfected with pYES1L-PEDV-EGFP and pCAGGS-PEDV-N was used to infect Vero CCL-81 cells. At 24 h post-transfection (hpt), a green fluorescence signal was observed in BHK-21 cells with DNA transfection, but not in the mock-transfected cells. Also, the typical CPE with EGFP expression was observed in Vero CCL-81 infected with the rPEDV-EGFP virus at 3 dpi, but no green fluorescence was observed for CPE caused by rPEDV infection. To further confirm the recovery of rPEDV-EGFP, N protein expression in red color was only detected by immunofluorescence assay in cells infected rPEDV-EGFP (Figure 6A). We also confirmed that the EGFP gene was expressed as an additional subgenomic RNA. As shown in Figure 6B, sequence analysis of subgenomic RNA suggested that the body transcriptional regulating sequence (TRS) of ORF3 mediated the jump of the subgenomic RNA expressing EGFP to leader TRS within 5’UTR. rPEDV-EGFP was serially passaged in Vero CCL-81 cells to check the stability of EGFP insertion. As expected, the EGFP gene was amplified and verified by DNA sequencing for P5 and P9 virus, suggesting the genetic stability of this reporter virus *in vitro* (Figure 4C).

The *in vitro* growth kinetics of rPEDV-EGFP were evaluated by the growth curves and plaque assay in Vero CCL-81 cells. As shown in Figure 6A, the growth kinetics of rPEDV-EGFP is similar to those of rPEDV, although its titers are about 0.5 logs lower than those of rPEDV at 48 and 72 hpi. Taken together, those results supported that ORF3 is not essential for PEDV replication.

## Discussion

The construction and manipulation of infectious cDNA clones for coronaviruses are always time-consuming and difficult for some laboratories without such experiences (Almazan et al., 2014). In this study, we generated an infectious cDNA clone of PEDV strain HM based on YAC/BAC system through one-step assembly and edited the genome *in vitro* using CRISPR/Cas9 technology and homologous recombination. The results suggested that this one-step assembly of PEDV infectious cDNA clone can be complete in 1 week from virus to infectious cDNA clone with superior efficiency, and the manipulation of PEDV genome with CRISPR/Cas9 technology is simpler and more rapid than the method of restriction enzyme digestion and ligation, providing an efficient platform for PEDV investigations.

The TAR cloning system in yeast has been used to generate many molecular virus clones in the past (Nikiforuk et al., 2016; Oldfield et al., 2017; Vashee et al., 2017; Thi Nhu Thao et al., 2020), especially the reverse genetics system of SARS-CoV-2 in such a short time (Thi Nhu Thao et al., 2020). The superior cloning efficiency of this yeast system was demonstrated in previous studies (Thi Nhu Thao et al., 2020). Taking advantage of the TAR technology, we generated an infectious cDNA clone of GII PEDV strain HM. Our results demonstrated the full functionality of the PEDV reverse genetics system generated using TAR technology. In line with the previous reports, the construction of PEDV infectious cDNA can be completed in 1 week without tedious cloning procedures. All of the colonies screened by colony PCR are positive (Figure 2D), suggesting the superior efficiency of yeast-based versus *E. coli*-based systems.

Based on previous studies of coronavirus reverse genetics, coronavirus genomes cloned in the high-copy number vector are usually unstable in *E. coli* because of their toxic effects on *E. coli* (Almazan et al., 2000; Yount et al., 2003; Scobey et al., 2013). To solve this unstable issue, several strategies were employed to establish coronavirus reverse genetics, including *in vitro* ligation of genomic cDNA fragments (Yount et al., 2003; Scobey et al., 2013), the use of BAC-based vectors (Almazan et al., 2000), vaccinia virus vectored CoV full-length cDNAs (Thiel et al., 2001), and the use of YAC-based vectors in yeast (Thi Nhu Thao et al., 2020). The first reverse genetics system of SARS-CoV-2 assembled in yeast using a YAC-based vector (pVC604) remained stable in yeast; however, their stability in *E. coli* was not evaluated (Thi Nhu Thao et al., 2020). Due to the lack of BAC elements in pVC604, the YAC-based infectious clone should be unstable in *E. coli*. The infectious clone plasmid extracted from yeast culture was linearized and used as a template for *in vitro* transcription to prepare SARS-CoV-2 genomic RNA. The recombinant SARS-CoV-2 was rescued *via* electroporation of *in vitro* transcribed viral genomic RNA. In our opinion, there are a few disadvantages of this reverse genetics: yeast plasmid extraction is more complicated, time-consuming, and expensive than bacterial plasmid extraction; for virus recovery, DNA transfection is simpler and cheaper than RNA transfection; The manipulation of the YAC-based infectious clone rely on the TAR in yeast. To improve our reverse genetics of PEDV, we assembled the PEDV infectious clone using the pYES1L vector containing YAC and BAC elements which facilitate stable plasmid replication in yeast and *E. coli*. A CMV promoter added at the 5′ end of the PEDV genome enabled virus rescuing *via* DNA transfection. The PEDV cDNA clone was manipulated *in vitro* through CRISPR/Cas9 cleavage and homologous recombination without relying on TAR in yeast.

In a previous study, CRISPR/Cas9 technology was applied to edit the PEDV genome with a BAC-based infectious cDNA clone (Peng et al., 2020), which was demonstrated to be a simple and rapid method. Deletions within ORF3 were detected in vaccine strains and many field PEDV strains (Cui et al., 2020; Lu et al., 2020b, 2021), suggesting that ORF3 may be non-essential for viral replication. Using reverse genetics systems, ORF3 was demonstrated to be not critical for PEDV replication and can be successfully replaced with reporter genes (Beall et al., 2016; Kaewborisuth et al., 2018; Peng et al., 2020). In the present study, using our YAC/BAC-based infectious cDNA clone, CRISPR/Cas9 nuclease cleavage and homologous recombination were combined to edit the PEDV genome *in vitro*. Consistent with the previous report (Peng et al., 2020), the recombinant PEDV expressing EGFP was generated within 1 week through the whole process from Cas9 nuclease cleavage, homologous recombination, transformation, plasmid preparation, and virus recovery. As expected, the expression of EGFP by rPEDV-EGFP was confirmed to be through the subgenomic RNA of ORF3. The reporter PEDV remained stable for at least 10 passages *in vitro*. With the same strategy, a recombinant PEDV expressing a secreted Gaussia luciferase was also generated (data not shown). Since virus replication can be monitored based on the reporter signal, these reporter PEDV viruses could be useful for the study of PEDV infection *in vitro* and *in vivo*.

In summary, taking advantage of the TAR technology in yeast, an infectious cDNA clone of GII PEDV was successfully established within 1 week. With this infectious clone, the CRISPR/Cas9 technology and homologous recombination were combined to rapidly edit the PEDV genome for the generation of a reporter PEDV which displayed similar replication properties to the parental virus.

## Data Availability Statement

The original contributions presented in the study are included in the article/supplementary material, further inquiries can be directed to the corresponding author.

## Author Contributions

YL, XW, and CS contributed to conception and design of the study. The experiments were performed mainly by YZ, CL, and YL, and some experiments were performed with the assistance of JH and CR. YZ, CL, and YL analyzed the data. YL wrote the manuscript. All authors contributed to the article and approved the submitted version.

## Funding

This project was supported by the National Natural Science Foundation of China (grant no. 31902254), the Jiangsu Agricultural Science and Technology fund (grant no. CX(21)3125), the Jiangsu Co-innovation Center for Prevention and Control of Important Animal Infectious Diseases and Zoonoses, and the Priority Academic Program Development of Jiangsu Higher Education Institutions (PAPD). YL is supported by the Scientific Research Foundation of Yangzhou University and the “LvYangJinfeng Program” of Yangzhou City.

## Conflict of Interest

The authors declare that the research was conducted in the absence of any commercial or financial relationships that could be construed as a potential conflict of interest.

## Publisher’s Note

All claims expressed in this article are solely those of the authors and do not necessarily represent those of their affiliated organizations, or those of the publisher, the editors and the reviewers. Any product that may be evaluated in this article, or claim that may be made by its manufacturer, is not guaranteed or endorsed by the publisher.
